# Nurses’ experiences of integrating the salutogenic perspective with person-centered care for older people in Swedish nursing home care: an interview-based qualitative study

**DOI:** 10.1186/s12877-024-04831-7

**Published:** 2024-03-18

**Authors:** Sofia Ehk, Sara Petersson, Atika Khalaf, Marie Nilsson

**Affiliations:** 1 Faculty of Health Sciences, Kristianstad University, SE-291 88 Kristianstad, Sweden; 2 Department of Nursing, Fatima College of Health Sciences, Ajman, United Arab Emirates

**Keywords:** Salutogenesis, Sense of coherence, Person-centered care, Older persons, Residential aged care

## Abstract

**Background:**

Even though there has been a cultural change within residential aged care to a more person-centered care, there remain improvements to be made for a more consistent way of working. Using a salutogenic approach along with person-centered care is a potential way to promote it. This study aimed to describe nurses’ experiences of combining person-centered care with a salutogenic approach at a nursing home for older people.

**Methods:**

Nine nurses, specially trained in salutogenesis and Sense of coherence, were individually interviewed using a semi-structured interview approach. Data was analysed through qualitative content analysis.

**Results:**

The nurses experienced that the residential aged care was improved by using salutogenesis and Sense of coherence as a complement to person-centered care. Core aspects of person-centered care were thereby promoted, as the resources of the older persons were emphasized, and aged care became more holistic. In addition to improved residential aged care, the results indicate that this manner of working also contributed to enhanced work satisfaction of the care personnel themselves.

**Conclusions:**

The results suggest that a salutogenic approach facilitates the implementation of person-centered care by focusing on the older persons’ resources and maintaining health. The organization needs to prioritize training staff in salutogenesis and person-centered care, as it supports working toward a common goal and benefits both the older persons and the staff.

**Supplementary Information:**

The online version contains supplementary material available at 10.1186/s12877-024-04831-7.

## Background

Traditionally, healthcare has focused on biomedicine using a reductionistic and pathogenic perspective. However, person-centered care has grown into an important complement to the more disease-oriented approach. As such, it means involving the patient to a larger extent and focusing on a more holistic care, which entails a recognition of the patient’s social, psychological, and spiritual aspects as well as the biological aspects [1]. Despite the claim of working in a person-centered way, studies show that care is not always person-centered, which can be due to obstacles such as lack of time, hierarchical organizations, and traditional approaches to clinical practice. This is also the case with the health care of older persons, adding a barrier of ageism to the other aggravating circumstances. Although there has been a cultural change within residential aged care to adopt more person-centered care as a way of guiding practice [2], much of aged care is not practiced in a systematic or consistent person-centered way [3]. Given that, the salutogenic approach may provide additional guidance for clinical practice and a way to increase person-centered care in aged care.

The International Council of Nurses ICN defines person-centered care from the aspects of making patients and their next-of-kin involved in the caring process [4]. Considering the patient’s resources and needs, the nurse must ensure that the person is at the center of the care. To ensure good care, the work can be done on a one-to-one basis person-centered approach, which is strengthened by respect for people, the older person’s right to self-determination, understanding the older person’s needs, and mutual respect between the caregiver and the older person [5]. Internationally, the World Health Organization has recognized the importance of a more holistic and people-centered approach to health care [6], and a cultural shift from a more traditional biomedical-focused approach to a more humanistic and person-centered approach can be seen within residential aged care [7, 8]. The shift in residential aged care towards a more humanistic and person-centered approach can be described as a movement away from the traditional biomedical model of care and towards a more holistic and individualized approach that prioritizes the emotional, social, and psychological needs of the residents. This shift involves a focus on creating a more homelike environment, promoting autonomy and dignity, and fostering meaningful social connections [9]. Recent research has highlighted the importance of understanding and evaluating the organizational culture in residential aged care facilities, as it is linked to the quality of care provided [10]. Furthermore, a recent meta-synthesis discussing this shift provided insights into the ongoing efforts to promote a more person-centered approach in residential aged care [11].

Previous studies have shown that person-centered care has improved residents´ psychological well-being and quality of life [5, 7], as well as their perceiving the quality of care [12]. Research also indicates that person-centered care may lead to both positive health outcomes and shorter hospital stays [13]. Adopting the perspective of the health care personnel, previous research has also indicated that nursing homes’ personnel experience the person-centered way of working as satisfactory [14–16] and contributing to their thriving at work [17].

However, there are challenges to implementing person-centered care. Such a challenge is the lack of a clear and agreed-upon definition, making person-centered care difficult to operationalize [18]. Another recognized challenge is the tendency to fall back to disease-orientated care, which means that focus is placed on the disease rather than the person [19]. Taken into a municipal care context, these challenges may be due to high staff turnover, lack of skills and resources and leadership without a mandate to change [20]. This is also pointed out by Liu et al. [21] who believe that it would be beneficial, and patient safety would be improved, if the working environment is ameliorated, the staff for nurses increased and sufficient support would be offered for nurses to focus more on patient care [21]. An ameliorated environment in older people’s residential care, as it relates to person-centered care, involves creating a setting that promotes independence, well-being, and a sense of community for the residents, while also supporting the job satisfaction and well-being of the care staff [22]. This is achieved through physical and social enhancements that prioritize the individual’s preferences, values, and desires, ultimately contributing to a more supportive and fulfilling care environment [23].

Being discriminated against due to one’s age, called ageism, is an obstacle to person-centered care in healthcare. It manifests itself as the older person being limited in different treatments due to age and being less involved in decisions about their own treatment than younger people [24]. This is also known by the fact that older patients hospitalized often do not receive information from the doctor about new drugs and treatment [25]. It is important to bear in mind that older patients are not a homogeneous group but individuals with different experiences and conditions [20, 26]. This means that older people want different things and many, despite chronic illnesses, want to be independent, take care of themselves and live in their own housing for as long as possible [25]. This is clarified in Global Goals Point 10 which is about an equal society and is based on the principle of equal rights for all and opportunities regardless of, for example, age [27].

Therefore, despite the advantages of working in a person-centered way there still remains the challenge of implementing and maintaining person-centered care in nursing homes. Challenges, such as lack of skills and resources along with a tendency to focus on the disease rather than the unique person, can be met by combining person-centered care with a salutogenic approach. This combination has been implemented in a nursing in Gothenburg, Western Sweden. The nursing home applies pronounced basic values of work in a salutogenic perspective. To examine the possible strength of combining person-centered care with a salutogenic basis of values, the aim of the study was to describe nurses’ experiences of person-centered care for older people in nursing home care combined with a salutogenic approach.

## Theoretical framework

The concept of salutogenesis was established by Antonovsky as early as 1979 as a complementary orientation to the previously established pathogenesis. While pathogenesis is concerned with risk factors for ill-health, salutogenesis focuses on aspects which actively promote health [28]. Antonovsky [28] believed that the salutogenic approach promotes health by using a person’s own resources. According to Antonovsky [28], the concept of health based on the salutogenic approach does not necessarily include the absence of disease, but the concept includes the person’s perceived health, the subjective experience.

In the nursing of older people, the salutogenic approach means that the older persons are seen as worthy human beings, with resources, a will to live, and an interest in their own well-being. There is also a belief that the person, despite increasing age and an aging body, still has the opportunity for both physical and mental strength [26]. The ICN Code of Ethics states that nurses must promote initiatives that meet the health and social needs of, above all, vulnerable groups, such as older people [29]. In order for the care of the older person to become salutogenic, Antonovsky [28] believes that it is necessary for the person to experience a sense of coherence (SOC). Antonovsky [28] suggests that three basic elements are required in order to experience SOC: comprehensibility, manageability, and meaningfulness. Comprehensibility regards people understanding themselves, their surroundings, their beliefs, and that others understand the person. Everything around and within oneself is perceived as understandable and the information is structured, coherent and clear. Manageability describes the extent to which a person’s resources are available when life is demanding. It may regard the person’s own inner resources, as well as informal support resources such as family, friends, or faith in God. According to Antonovsky [28], meaningfulness regards how a person views challenges given in life, if they are something that enriches life, or if they are seen as heavy burdens.

## Methods

### Study design

This study was conducted with a qualitative method and a deductive approach, using semi-structured interviews. Deductive qualitative analysis typically involves analyzing data according to an existing theoretical framework, and it is not necessarily aimed at ‘testing’ the theory, but rather at applying theory to the data to test or confirm its applicability [31] which has been applied in our data analysis.

### Context

The participating nurses were selected from Tre Stiftelser in Gothenburg. Tre Stiftelser consists of three different nursing homes where about 360 older people live and receive care. The foundation works on the basis of salutogenesis where the starting point is the health of each individual. The care is provided based on the three components found in SOC; namely, meaningfulness, comprehensibility and manageability. All personnel at the Tre Stiftelser underwent a training course in salutogenesis and SOC. In addition, a person-centered approach is applied which includes the older person’s condition, wishes, and needs.

### Sample

A convenient sampling technique was applied [32] to recruit participants. The participating nurses were all registered nurses, of which two were post-graduates and trained as geriatric nursing specialists. After the approval of the unit managers, nurses were recruited through posters in the staff rooms. The inclusion criteria were a registered nurse who had worked for more than 6 months at Tre Stiftelser. Information on the poster consisted of the study’s aim, the inclusion criteria for participation in the study, and the contact information of the principal investigator. When the poster had been up for four weeks, 10 nurses sent an email to register their interest in participating, but only nine nurses were interviewed as one did not confirm the day and time for the interview. The average age of the participants was 36 years, and the average work experience was eight years. The information sheet was sent to nurses prior to the interviews. The participants chose the day and time for the interview.

### Data collection

An interview guide (see Supplementary Material 1) was developed and tested through two pilot interviews before the study began. The results of the pilot interviews showed that the question guide worked well, and no amendments were made. The pilot interviews were included in the analysis, as they had depth and answered the aim of the study.

The participants in the study received written information before the interviews were booked and orally before the interviews started. The information concerned the purpose of the study and that participants could cancel the interview at any time. Written consent was obtained, and the participants were given the opportunity to ask questions before the interview began. All interviews were conducted digitally via Zoom but recorded via Dictaphone. Saturation was reached after seven interviews, and two additional interviews were conducted to ensure that no new information appeared. The interviews lasted about 40 min in average.

### Data analysis

Transcribed material was processed through content analysis based on Graneheim and Lundman [33]. Before the analysis began, the transcribed material was read individually several times to create an understanding of the material. The material was then analyzed by three authors separately, and a manifest analysis initiated the whole analysis process [33, 34]. A manifest analysis approach involves staying very close to the text and taking the meaning of the words at face value [35]. In our analysis, we focused on the explicit and observable aspects of the data, such as the words and phrases used by the participants, to identify patterns and themes related to person-centered care in older people residential care. The authors then met digitally via Zoom and discussed the findings before agreeing on the meaningful units that corresponded to the aim. These were then condensed, and codes were created, sorted into subcategories, and finally into four main categories. The results of the study were then presented, clarified, and strengthened by quotes from participating nurses. The quotes were corrected for spelling. An example of the analysis is presented in Table 1.

**Table 1:** Example of the analysis procedure

Meaning unit	Condensed meaning unit	Code	Sub-category	Category
The salutogenic values, the sense of coherence, it should permeate person-centered care and it becomes more concrete. It becomes clearer when we reflect on whether this person has comprehensibility, manageability, and meaningfulness. These are the tools that can contribute to person-centered care.	The salutogenic values and sense of coherence should permeate person-centered care, which becomes clearer. Reflecting on a person’s comprehensibility, manageability, and meaningfulness is a tool to contribute to person-centered care.	Securing sense of coherence contributes to person-centered care.	The integration of salutogenesis with person-centered care.	The holistic approach in person-centered care is supported by the salutogenic perspective.

### Ethical consideration

In adherence with the recommendations of the Helsinki Declaration [36], the participants have given written informed consent by responding to the invitation email and agreeing to participate in the study. In addition, verbal consent was recorded during the interview session after the participants were given oral information before and during each interview. The participants were thereby informed that participation in the study was voluntary and that they could cancel their participation at any time during the course of the study without stating the reason. Information was also given regarding how data regarding person and place are treated confidentially. Data were decoded by changing the participant’s name to reduce the risk of identification [36]. The study received ethical approval from the Faculty of Health Sciences at Kristianstad University (D1DF:2).

Some risks have been identified with participation in the study. One risk is that the nurses at Tre Stiftelser may have felt obliged to participate in the study as one of the authors is a former colleague. Volunteering can then be questioned, but at the same time, all participants have given their consent before the interviews. Another risk is that participating nurses may have experienced stress due to a strained work situation, at the same time as they wanted to participate in the study [36].

Additionally, the professional experience of the authors was that a salutogenic approach might make it easier for nurses to provide person-centered care. However, the authors discussed their pre-understanding and summarized this before the interviews began to minimize its possible influence on the analysis and results presentation.

## Results

The use of a salutogenic perspective in combination with person-centered care is experienced by the nurses as improving residential aged care as well as contributing to the work satisfaction of the care personnel themselves. Hence both the older persons and the care personnel benefit by this way of working. By using a salutogenic perspective along with person-centeredness, a focus on the resources of the older persons is emphasized, as is using a holistic perspective. Person-centered care is made concrete by the salutogenic approach, manifested in comprehensibility, manageability, and meaningfulness. The findings are represented in three categories (see Table 2).

**Table 2:** Nurses’ experiences of person-centered care for older people in nursing home care, combined with a salutogenic approach

Categories	Sub-categories
Emphasized focus on the resources of the older person	Participation promotes a sense of coherence
	The older person’s self-determination
	Communication creates a partnership
The holistic approach in person-centered care is supported by the salutogenic perspective	The integration of salutogenesis with person-centered care
	The coexistence of salutogenesis and pathogenesis in person-centered care
Integrating the salutogenic perspective with person-centered care enhances the work satisfaction of the care personnel	Training in salutogenesis and SOC promotes the assignment of providing person-centeredness.
	The values of the organization correlate with the essential values of the nurses

### Emphasized focus on the resources of the older person

This category concerns the nurses’ focus on the resources of the older person and entails three sub-categories: participation promotes a sense of coherence; the older person’s self-determination; and communication creates a partnership.

By encouraging the older persons to participate in their own care, the nurses believed a SOC was enhanced for the older persons. Participation, as in codetermination, was thereby a way to involve and make use of the resources of the older person. Nurses described that enhanced SOC as influencing the older people. For example, by allowing the older persons to decide which time it worked best to dress their wounds, the participation in their own care improved their understanding of the situation and their needs. As a result, their comprehensibility was improved. The nurses subsequently planned according to the older persons’ wishes, and their manageability was thereby also strengthened. The nurses expressed that this contributed to empowerment and to increased meaningfulness for the older person.

Nurses also described that in using a salutogenic perspective, and thus focusing on resources, the self-determination became a central part of the care, in line with person-centered care. Nurses would not control the direction of care. The nurses’ experience was that they could offer their help, but it was still on the older persons’ terms, and their opinion was considered important. The co-determination sometimes became less important than the self-determination of the older person. The nurses described situations when they chose to listen to the older person and prioritize his or her wish, even when it meant standing up against the doctor:

*When the person says “I do not want to do this anymore, I have lived my life, I do not need to prolong it, and I want to end the treatment” then I think it´s about listening to what is required.* (Nurse No. 4)

The dialogue with the older persons created a partnership, which coincided with the focus on resources in both the salutogenic approach and person-centered care. Through the communication, the nurses gave information regarding, for example, which blood samples to take and their purpose, and the older people had the opportunity to ask questions and together they could decide on the course of action. In this way, older persons became involved in their own care and could then decide if they wanted to participate or not. The nurses described it as the older person becoming co-determinant in their care, and they became partners in the care process.

Communicating with older persons also meant that nurses obtained knowledge and awareness of what the specific person considered important, the experiences and the feelings. The dialogue became the means on which the partnership was created, and a compassionate and respectful relationship was built:

*When I sit down and talk to […] and they want to tell me things and share their feelings, their lives… it is a confidence and a trust.* (Nurse No. 5)

Therefore, by becoming partners, not only the resources of the nurse were guiding the care but also the resources of the older persons.

### The holistic approach in person-centered care is supported by the salutogenic perspective

The nurses described that by using a salutogenic care model the holistic approach in person-centered care was emphasized. The holistic approach entailed an integration of different perspectives, which were manifested in two subcategories: the integration of salutogenesis with person-centered care; and the co-existence of salutogenesis and pathogenesis in person-centered care.

According to nurses’ experiences, the salutogenic approach supported the person-centered care, as it became more pronounced when regarding the care according to the concepts of comprehensibility, manageability, and meaningfulness:

*The salutogenic values, the sense of coherence, it should permeate person-centered care and it becomes more concrete. It becomes clearer when we reflect on whether this person has comprehensibility, manageability, and meaningfulness. These are the tools that can contribute to person-centered care.* (Nurse No. 5)

The salutogenic approach was also described as enhancing the quality of life. An example of this was when the nurses understood how important it was for the older persons’ well-being to take part in social activities and went out of their way to support it. Instead of accepting that some older people declined to partake, they assumed the perspective of the older person, that is, the insider’s perspective, and barriers such as incontinence were discovered and could be met by appropriate aids. So, by dealing with the older persons’ feelings of insecurity on account of their fear of leakage, the nurses helped to make the situation manageable. As a result, the older persons’ everyday life became more meaningful and their quality of life improved. This was made possible by using an insider’s perspective, which in itself entails a holistic perspective.

The salutogenic approach and person-centered care was experienced as intertwined and interrelated, as one nurse:

*We look at another whole in the salutogenic perspective… we weave person-centered care into the salutogenic ones automatically; I think it is difficult to be salutogenic if you do not believe in the person-centered [care]… they are connected.* (Nurse No. 4)

The holistic aspect in person-centered care was also supported by using both the salutogenic perspective as coexistent with the pathogenic perspective. The pathogenic approach was needed for the older persons to be able to live an active life, and the nurses pointed out that the older persons’ illness must be taken care of for them to experience well-being and quality of life. As such, the pathogenic and salutogenic approach complement each other and contribute to a holistic approach, and both are used in person-centered care.

An example of this was when an older person with pain received pain relief before a gymnastics session to be able to participate. The pain needed to be taken care of first, i.e., the pathogenic intervention, but it was complemented by the ambition to facilitate the physical activity as it was salutogenic. Salutogenic and pathogenic approaches were conducted with a conscious of their complementary relationship:

*Emanating from my profession as a nurse, I believe that working in a salutogenic approach means having to… keep what’s ill in check in order to notice all the resources that the person has.* (Nurse No. 9)

### Integrating the salutogenic perspective with the person-centered care enhances the work satisfaction of the care personnel

The findings indicated that not only the residential aged care was improved by the integration of the two approaches, but also the work satisfaction of the nurses. This was manifested in two subcategories: training in salutogenesis and SOC promoted the assignment of providing person-centered care; and the values of the organization correlated with the essential values of the nurses.

All personnel at the nursing home underwent a training course in salutogenesis and SOC, and this course was considered by the nurses as a vital part of conducting a care model that integrated the salutogenic perspective with person-centered care. They believed that this training was of great help in daily work and ensured that everyone in the organization was working towards the same goal, from nurses and facility caretakers to the managers. Furthermore, the nurses believed that meaningfulness was enhanced when all staff knew what they were working towards, and this enhanced their work satisfaction. One nurse expressed it in terms of creating SOC for the personnel as well:

*[knowing that we all have the same goal, i.e. providing good care for the older person] makes it easy. I think […] it creates SOC also for the employee, everyone knows the focus, everyone knows why I am at work.* (Nurse No. 2)

Working in a nursing home with the pronounced values of salutogenesis also signified an agreement with the essential values of the nurses themselves. This was described by the nurses as promoting feelings of fulfillment and satisfaction. However, supportive management was experienced as a prerequisite to creating such a work situation. They experienced an understanding from the management that caregiving takes time, and a support that allowed putting the older persons first, even when it meant a more time-consuming care. For example, the management encouraged having a cup of coffee with the older person, creating a more enjoyable life for both the nurse and the resident:

*I believe that with the salutogenic approach you can create a life that is pleasant, or how do you say… a better life. Where you can continue to be yourself.* (Nurse No. 1)

## Discussion

The aim of the study was to describe nurses’ experiences of person-centered care for older people in nursing home care, combined with a salutogenic approach. Our main findings reflect the nurses’ experiences with an emphasis on the older persons’ resources, the perception that the holistic approach in person-centered care is supported by the salutogenic perspective and that integrating the salutogenic perspective with person-centered care could improve the nurses’ work satisfaction. The experiences of the nurses could be described as the result of person-centered care alone. However, nurses described their experiences as emphasized by adding the salutogenic perspective to person-centered care.

In the current study, the nurses’ emphasis on the older persons’ resources was described in how they tried to promote a sense of coherence through participation which they perceived was a core practice in person-centered care. Participation was created through dialogue, listening to older persons, and providing space for them to express themselves. Participation was defined by Sahlsten et al. [37] as being engaged, involved, and participating in the context at the same time as the nurse invites the persons to participate in their care. Both parties actively participate, and participation means that the people are involved in decision-making concerning their own care [38]. The findings of the study showed that both the nurse and the older persons needed to actively participate in order to create participation. The results of the study also showed that co-determination that arose through the participation of older persons led to SOC. Antonovsky [28] points out that SOC is about how comprehensibility, manageability, and meaningfulness affect the lives of older people. In addition, the Swedish Nurses’ Association [38] states that nursing should be based on persons’ participation and that care should be person-centered. Furthermore, the competency description for special nurses in the care of the older persons refers to identifying and working based on the needs and resources [38], which strengthens the result of our study. Hence, participation is an important building stone in providing person-centered care for the older people. Nurses need therefore to take all possible measures and plan active interventions to promote older persons’ participation, increase their influence, and build their capacity to participate in their own care [39].

Another reflected experience in the current study was the holistic approach in which nurses supported person-centered care through the salutogenic perspective. For example, SOC was mentioned by the nurses as a valuable tool towards practicing a holistic person-centered care. They meant that the SOC tool promoted comprehensibility, manageability, and meaningfulness in concretizing the care provided. Zhao et al. [40] showed that healthcare professionals may have difficulty seeing the whole person, which could lead to important information about the person’s goals sometimes not being noticed. Similarly, Arakelian et al. [30] showed that person-centered care was important for hospitalized persons to be seen as unique individuals. Furthermore, they believed that it is important that their personal wishes are considered in the care provided [30]. The results of the current study are reinforced by what Arakelian et al. [30] point out by explaining the importance of the nurse considering and seeing the entire older person. This is the essence of being able to reach a person-centered approach. To implement holistic, person-centered care, nurses need to be informed about the older persons’ situation, their expectations of the care provided, and their personal wishes and resources. Studies have shown that a presence of a strong SOC in the older person is associated with better physical, social, and mental health [41] which is linked to good perceived health. Furthermore, healthy aging means having something meaningful to do and a balance between capacity and challenges for the older person [42]. Our study shows that person-centered care is supported through a salutogenic approach and focusses on maintaining health and creating quality of life. This is done through activities and based on what the older persons wish. Antonovsky [28] believes that in salutogenic approaches, health-promoting measures are sought that facilitate people’s health by taking advantage of and supporting their own resources. By ensuring health and belief in one’s own ability, as well as valuing perceptions and experiences, health is promoted [28]. This strengthens the results of our study as the context is important for person-centered care. It will thus be easier to work person-centered based on a salutogenic context.

Furthermore, the study showed that nursing was experienced as a pleasant practice due to the organization’s choice of values. The nurses reflected on the congruence between the organization’s values (salutogenesis) and the core code of nursing (person-centered care). This led to increased feelings of satisfaction with the work tasks and fulfillment of their commitment towards caring for older people. One common component with Sharma et al. [43] findings was related to the caregiver’s behavior and the organizational support required for person-centered care. The conclusions from Sharma et al. [43] show that healthcare providers and organizations need to facilitate person-centered care. There also needs to be shared decision-making and meaningful participation to improve the healthcare system [43]. This reinforces the result in our study, in which the experience was that a higher motivation is created among the staff when the organizational values reflect the nursing values. Furthermore, the study showed that if everyone knew why they were at work, SOC was also created among the employees. Therefore, it is recommended, based on our findings, that an organization to communicate clear goals and values to enhance the understanding of the vision of the organization’s vision and thus the purpose of their own work. In addition, our results show that the work towards person-centered care and patient contact were among the most rewarding parts of being a nurse. Nurses also reported that the organization’s salutogenic value positively affected their nursing care. We believe therefore that the organization has an implicit role in creating a work environment that might affect how good the quality of care the older persons receive.

### Methodological aspects

To strengthen the trustworthiness of the current study, an appropriate method was chosen (a qualitative method) to answer the research question. An example of the analysis procedure was provided in a table (to increase the credibility of the study results), quotes from the interviews were used (to substantiate the results), the study methods were described as accurately as possible (to enhance the verifiability of the study), and more than one researcher has been involved in the analysis work (to increase the credibility of the study) [44].

A qualitative method with a deductive approach was chosen (to correspond to the phenomenon studied). The data collection, the data analysis, and the results reflect the perspectives of nurses, who were educated in the salutogenic perspective and responded to questions guided by the salutogenic approach (the interview guide), which complies with the deductive approach [31].

In addition, two pilot interviews were conducted to see if the interview guide corresponded to the purpose of the study, which it did. After the first pilot interview, it was discovered that the author, who previously worked at Tre Stiftelser, asked fewer follow-up questions. This was linked to the pre-understanding of the study’s context and theoretical frame of reference, the authors then jointly decided that the second author would conduct the remaining interviews. This meant that the author with the pre-understanding of the context was aware of her pre-understanding. If this had not been discovered, the trustworthiness of the study could have been questioned and it is not certain that the material provided the same amount of data. Another aspect that may have affected the amount of data was that the interviews took place digitally via Zoom and not face-to-face. However, the interviewing author actively listened, which meant that relevant follow-up questions were asked [32].

To strengthen the transferability of our study results based on the recommendations by Graneheim et al. [34], we have provided a thorough description of the chosen context, a relevant background of the chosen subject, a theoretical frame, a summary of existing evidence, a careful description of the selection and recruitment processes, and an example of the analysis process. By describing all these procedures, the reader is given the basic requirements to be able to assess whether the results of the current study are transferable to their contexts and situations. Our conclusion in this regard is that the results are only transferable to contexts in which the salutogenic approach is adapted as an inspiring or guiding framework.

## Conclusions

The result of the study showed that the salutogenic approach is important and facilitates the implementation of person-centered care for the older persons. For care to be person-centered, the older persons need to be involved in their care, which can be facilitated by using a salutogenic approach, emphasizing the older persons’ own resources. The study also showed that care is facilitated when the entire organization works towards a salutogenic approach, with the older person at the center. Thus, the organization is recommended to prioritize training staff on combining the approaches of person-centered care and salutogenesis. Furthermore, health care providers’ work satisfaction can be enhanced by applying salutogenesis. Suggestions for further research based on the results of the study is a comparative study where nurses are interviewed based on their experience of person-centered care without a salutogenic context.

### Electronic supplementary material

Below is the link to the electronic supplementary material.

Supplementary Material 1

## Data Availability

The datasets used and/or analysed during the current study are available from the corresponding author on reasonable request.

## Abbreviations

ICN: International Council of Nurses
SOC: Sense of Coherence
