# Supplementary material for: Nurses’ experiences of integrating the salutogenic perspective with person-centered care for older people in Swedish nursing home care: an interview-based qualitative study
Source: BMC Geriatr. 2024 Mar 18;24:262. doi: 10.1186/s12877-024-04831-7 (PMC10946094; doi:10.1186/s12877-024-04831-7)
Supplement: Supplementary file 1 — Supplementary Material 1 [file 12877_2024_4831_MOESM1_ESM.docx]

**Interview guide**

**Definition of salutogenic values:** A care which emanates from the starting point:

What enhances a person’s well-being?

**Background information**

Would you please tell me…

How old are you?

How long have you been working as a registered nurse?

Have you attended any specialist training?

How long have you been working at Tre Stiftelser?

Did you have any previous care experience before your training to become a nurse?

Have you worked in any other care context as a registered nurse before starting your work at Tre Stiftelser?

**Context (Tre Stiftelser)**

What would you say is the difference between the nursing home care at Tre Stiftelser and other nursing homes?

**Salutogenesis**

How would you describe salutogenic values?

The salutogenic approach, what does it mean to you, in your role as a nurse?

Can you give me some examples of how you work in a salutogenic approach?

Would you please describe a situation in which you would say the care became salutogenic for the older person?

Would you describe a situation in which the care was not characterized by a salutogenic approach? What could have been done for the care to be salutogenic instead?

What would you say is the main difference between a salutogenic approach and a pathogenic approach?

Which advice would you like to give to a nursing home that wishes to change its care and include a more salutogenic approach to caring?

**Person-centered care**

What does person-centered care mean to you in your role as a nurse?

When, would you say, do you perform person-centered care as a nurse?

Would you describe a situation in which the care becomes person-centered for the older person?

Would you describe a situation in which the care was not person-centered? What could have been done for the care to be person-centered instead?

**Combined salutogenesis and person-centered care**

Would you say that the salutogenic values have an impact on the person-centered care for the older person, and if so, in what ways?

**Prompting questions to deepen the interview are:**

Would you please tell me more about…

What do you mean by…
